# Pathways to Participation – Understanding Barriers and Enablers to Participation in Dementia Research

**DOI:** 10.1002/alz70861_108736

**Published:** 2025-12-23

**Authors:** Lukas Duffner, Soraya Moradi, Ana Diaz, Dianne Gove, Angela Bradshaw, Jean Georges

**Affiliations:** ^1^ Alzheimer Europe, Senningerberg Luxembourg

## Abstract

**Background:**

The ongoing recruitment crisis in dementia research highlights the importance of understanding public attitudes and experiences related to study participation. This scoping review aimed to examine current evidence on the factors that hinder or support the recruitment and continued involvement of people in dementia research.

**Method:**

A scoping review was conducted using PubMed and PsycINFO databases, guided by the Arksey and O’Malley methodological framework. A pilot‐informed keyword strategy was used (detailed protocol: osf.io/n8j4u). Titles, abstracts, and full texts were independently screened by two reviewers, and data were extracted based on predefined criteria. Identified barriers and facilitators were organized into thematic categories. Additionally, insights were gathered from a panel of people living with dementia and their carers, who provided reflections on the review findings.

**Result:**

A total of 45 studies, involving 112,011 participants (average age 69.3 years; 64.8% female), were included in the synthesis. Most studies were based in the United States and focused on clinical dementia research, with a predominant focus on recruitment rather than long‐term retention. Seven overarching themes emerged as barriers: (1) mistrust; (2) fears, worries and concerns; (3) beliefs, awareness, and attitudes; (4) logistical and practical challenges; (5) characteristics of the study itself; (6) informational barriers; and (7) the role of carers and support networks. Enablers included personal motivations (e.g., desire to help others, interest in gaining health insights) and external facilitators (e.g., tailored engagement strategies, logistical accommodations).

**Conclusion:**

Despite existing knowledge gaps, many of the identified barriers to participation in dementia research are addressable. Strategic interventions targeting these modifiable issues may improve both recruitment and retention, enhancing inclusivity and effectiveness of future dementia studies.

